# Low Temperature Sensing Properties of a Nano Hybrid Material Based on ZnO Nanotetrapods and Titanyl Phthalocyanine

**DOI:** 10.3390/s130303445

**Published:** 2013-03-13

**Authors:** Nicola Coppedè, Marco Villani, Roberto Mosca, Salvatore Iannotta, Andrea Zappettini, Davide Calestani

**Affiliations:** Istituto dei Materiali per l' Elettronica ed il Magnetismo (IMEM), Consiglio Nazionale delle Ricerche (CNR), Parco Area delle Scienze 37/A 43124 Parma, Italy; E-Mails: nicola.coppede@imem.cnr.it (N.C.); mvillani@imem.cnr.it (M.V.); mosca@imem.cnr.it (R.M.); iannotta@imem.cnr.it (S.I.); zapp@imem.cnr.it (A.Z.)

**Keywords:** gas sensor, ZnO nanostructures, phthalocyanine, NO_2_, room temperature

## Abstract

ZnO nanotetrapods have recently been exploited for the realization of high-sensitivity gas sensors, but they are affected by the typical drawbacks of metal-oxides, *i.e.*, poor selectivity and a relatively high working temperature. On the other hand, it has been also demonstrated that the combined use of nanostructured metal oxides and organic molecules can improve the gas sensing performance sensitivity or selectivity, even at lower temperatures. A gas sensor device, based on films of interconnected ZnO nanotetrapods properly functionalized by titanyl phthalocyanine (TiOPc), has been realized in order to combine the high surface to volume ratio and structural stability of the crystalline ZnO nanostructures with the enhanced sensitivity of the semiconducting TiOPc molecule, especially at low temperature. The electronic properties of the resulting nanohybrid material are different from those of each single component. The response of the hybrid nanostructure towards different gases has been compared with that of ZnO nanotetrapod without functionalization in order to highlight the peculiar properties of the hybrid interaction(s). The dynamic response in time has been studied for different gases and temperatures; in particular, an increase in the response to NO_2_ has been observed, even at room temperature. The formation of localized p-n heterojunctions and the possibility of exchanging charge carriers at the hybrid interface is shown to be crucial for the sensing mechanism.

## 1. Introduction

Zinc oxide (ZnO) is one of the most used metal oxides for gas sensing applications, together with tin oxide (SnO_2_), titanium oxide (TiO_2_), tungsten oxide (WO_3_), *etc.* But, among these, ZnO is surely the only material that can be obtained in several nanostructure forms with different morphologies [1–4]. Nanotetrapod (TP) is one of its typical crystalline morphologies, consisting in four needle-shaped “legs” that are connected at one common end and arranged as axes of a tetrahedron, which forms highly porous films that have been recently exploited for the realization of high-sensitivity gas sensors [5,6]. However, these sensors are affected by some of the typical drawbacks of metal-oxides, *i.e.*, a poor selectivity and a relatively high working temperature (often above 200 °C) [7].

These intrinsic limitations of metal oxides have given rise to new research approaches such as, for example, those focusing on innovative hybrid nanostructured materials, based on the interaction of organic-inorganic materials and on the combination of the specific properties of both classes of material [8]. It has been demonstrated that the combined use of nanostructured metal oxides and organic molecules can help to overcome some of these issues [9,10], also enhancing the sensitivity and/or selectivity towards specific gases at lower temperatures, where organic materials are usually more chemically active than oxides.

Metal-phthalocyanines (MPc) are among the most studied class of molecules for gas sensing applications, since their electric properties strongly depend on the chemical species in the surrounding atmosphere [11]. MPc films present a p-type semiconductor behavior and their electronic properties, due to the delocalized π-electron system, present quite interesting values [12]. They have stable molecular structures and can be tailored by chemical engineering, to modify their optical and electronic properties. On the other hand, like other organic molecules, MPc have some limitations as gas sensors, such as a relatively low solid-state stability and slower response and recovery [13] than that typical of metal-oxides operating at high temperature.

Organic–inorganic hybrid nanostructures are receiving considerable attention aimed at developing “smart” materials with new tailored properties. Gas sensing is one of the most interesting applications where the control on the properties of the nano-hybrid material may overcome the unavoidable limitations of the devices. The interaction between nanostructure of commonly used metal oxides and organic molecules may give rise to peculiar transduction effects, selectivity enhancements, extended working temperature ranges or, in general, improved electric properties of the material [8,14]. Moreover, the possible tailoring of the organic molecule structures allows researchers to design different functionalizations with different selective properties, paving the way to nanostructured gas sensor devices that can benefit from new sensitivity and selectivity properties [9].

In this work we realized gas sensor devices based on films of interconnected ZnO-TPs properly functionalized by titanyl phthalocyanine (TiOPc). The aim was to combine the high surface to area ratio, the structural stability and the good electronic transport properties of the crystalline ZnO nanostructures with the possible enhanced sensitivity or selectivity due to the semiconducting TiOPc molecule, especially at low temperature. The formation of localized p-n heterojunctions and the possibility of exchanging charge carriers at the hybrid interface are expected to strongly affect the electronic properties of the resulting nanohybrid material that, as anticipated, resulted to be different from those of each single component. We tested the responses of the obtained ZnO-TiOPc hybrid nanostructure, comparing it with those of ZnO-TPs without functionalization in order to highlight those cases in which different behavior was observed. The peculiar properties of the hybrid sensor towards different gases have been tested, as a function of the working temperature, from RT up to 300°C. The dynamic response *vs.* time, temperature and relative humidity (RH) have been studied and, in particular, a very high response towards NO_2_ has been observed, even at room temperature.

## 2. Experimental Section

**Figure 1.**
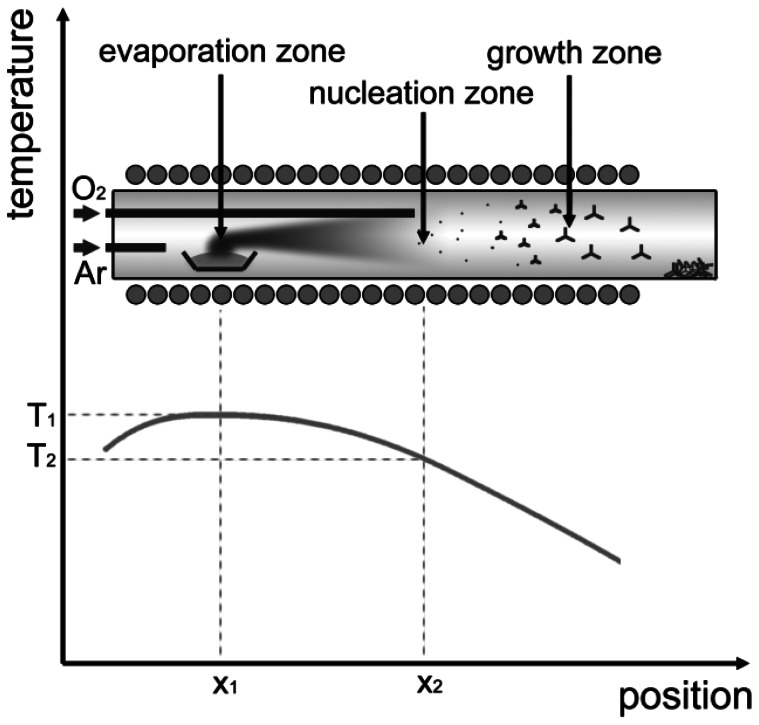
Vapour Phase Deposition apparatus.

The growth of ZnO-TPs has been realized by a properly optimized vapor phase growth technique [15], inside a tubular furnace, provided with two separate gas inlets for argon and oxygen. A scheme of the growth system is shown in Figure 1, together with the typical temperature profile. Temperature profile, gas inlets at different positions and gas flow define three zones inside the reactor, *i.e.*, “evaporation” “nucleation” and “growth” zones. In the first part of the tube (the “evaporation” zone) source material (99.999% pure Zn powder) is kept at a higher temperature (about 700 °C) with respect to the downstream (“nucleation” and “growth”) zones. Zn vapors are transported from the “evaporation” zone by an argon flow (100 sccm) to the “nucleation” region, where direct reaction with oxygen (flowing at about 5 sccm) promotes the formation of a large amount of solid ZnO nuclei. These nuclei floating in the carrier gas towards the “growth” zone develop in the form of tetrapod-shaped nanocrystals thanks to the high supersaturation conditions up to when they reach the coldest part at the end of the furnace, where they deposit.

The collected ZnO-TPs can be suspended in isopropanol and then deposited at room temperature on different substrates. In this case, the obtained ZnO nanostructures are deposited on a device—prepared alumina substrates with gold contacts and a Pt stripe for heating the sensor if required. The amounts of ZnO-TPs obtained in our laboratory-scale reactor are sufficient to prepare hundreds of sensors.

The prepared substrates with contacts and ZnO nanostructures are then inserted in another tubular furnace to deposit the functionalizing TiOPc by a Physical Vapor Deposition (PVD) process. TiOPc powder is set in the center of the tubular furnace, with the substrate downstream at lower temperature. The quartz tube is evacuated through a pumping system to 10^−5^ mbar and TiOPc powder is then heated at about 480 °C. The temperature on the device-ready substrates, on the contrary, does not exceed 100 °C. The structure and morphology of the obtained film was studied by Scanning Electron Microscope (QUANTA ESEM FEG 250, FEI Company, Eindhoven, The Netherlands) and absorption measurements.

**Figure 2.**
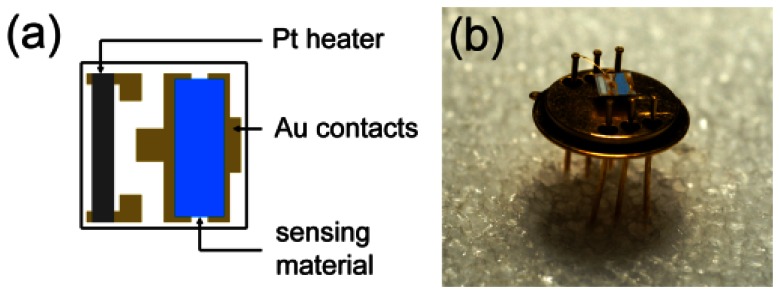
(**a**) Sketch of parallel sensor heater and contacts geometry on alumina substrate; (**b**) image of the sensor bonded in floating configuration on a TO8 package.

The gas sensor is then completed by removing ZnO-TPs and TiOPc from undesired regions of the substrate, like the heater and the bonding pads, leaving the coupled nanostructure only on the top of the two main contacts (Figure 2(a)). The sensor substrate is finally mounted on a TO8 package by gold wire bonding (Figure 2(b)). Sensors with only ZnO-TPs and TiOPc were also prepared for comparison.

The obtained gas sensors are then tested by using the flow-through technique [16], under a constant flow of 500 mL/min of the desired mix of air, humidity and test gas. Sensor chemoresistance is studied by the volt-amperometric technique at constant voltage (DC) of 5 V. Dynamic responses were obtained using different concentrations of test gases (ethanol, NO_2_) as a function of sensor temperature, ranging from room temperature (RT) up to 200 °C, and relative humidity (RH), ranging from 0% to 75% (measured at 25 °C).

## 3. Results and Discussion

**Figure 3.**
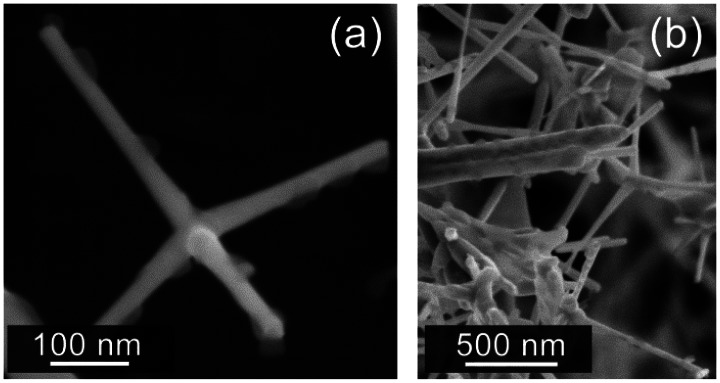
SEM images of ZnO-TPs+TiOPc samples: (**a**) a single ZnO-TP with TiOPc spots; (**b**) a lower magnification image of the typical network formed by these nanostructures

Figure 3 shows SEM images that clearly illustrate the morphology of the obtained coupled ZnO-TPs+TiOPc samples. ZnO-TPs with average “legs” thicknesses of 30–100 nm and length of 0.2–2 μm are functionalized in a non-continuous way by TiOPc spots, leaving part of the ZnO surface directly accessible to the gases.

**Figure 4.**
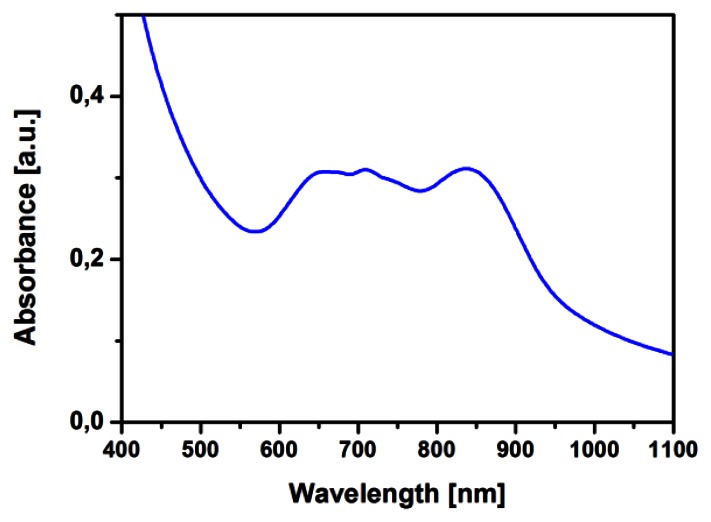
UV-Visible absorption spectra of TiOPc film deposited by Vapor Phase Deposition, showing the typical phase II peaks.

The TiOPc structure has been characterized by means of absorption measurements. In Figure 4 the UV-visible absorption spectrum of a TiOPc film deposited on glass is shown. The UV-visible spectra display typical Q band peaks of TiOPc and the presence of a peak at 850 nm, together with the separation in the band from 650 nm to 850 nm typical of a phase II TiOPc, as demonstrated in the literature [17]. The presence of an ordered crystalline phase for TiOPc usually provides a higher stability and improved conduction properties for the devices, when compared to the amorphous phase. The sample for this measurement has been prepared on a quartz substrate together (side-by-side in the reactor) with the alumina sensor-ready substrate, so in the same position, temperature and pressure conditions of the one deposited on ZnO-TPs for gas sensing tests.

Gas sensing properties have been compared between a sensor made with ZnO-TPs only and a sensor made with TiOPc-functionalized ZnO-TPs in order to better evaluate the effectiveness of the functionalization.

**Figure 5.**
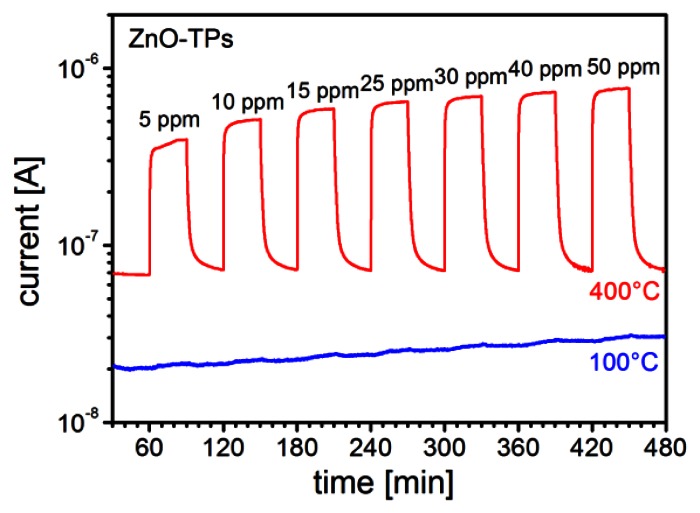
Comparison of the dynamic responses of a sensors with ZnO-TPs only towards different concentrations of ethanol at 100 °C and 400 °C (RH 30%).

**Figure 6.**
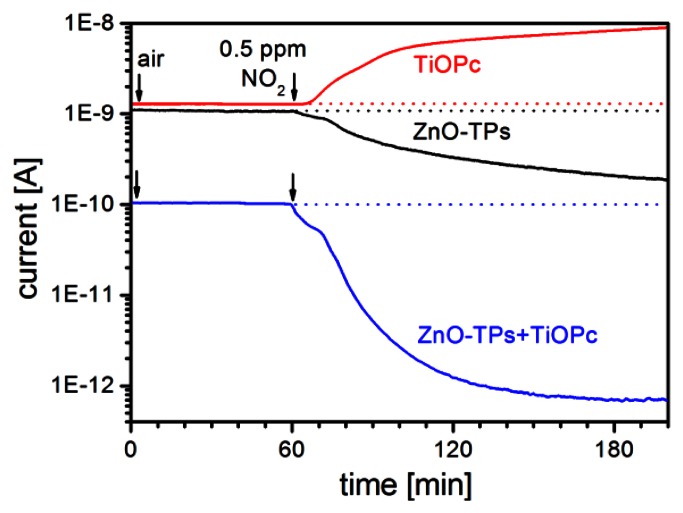
Comparison of the dynamic response of ZnO-TPs, TiOPc, and ZnO-TPs+TiOPc sensors towards 0.5 ppm of NO_2_ at room temperature (RH 30%).

In Figure 5 the response to different ethanol concentrations (from 5 ppm to 40 ppm) for a gas sensor based on ZnO-TPs without any functionalization is reported for two different temperatures. It is possible to see there that sensor works well at the typical working temperature of 400 °C, while the response is very low when operating at 100 °C. This is a typical behavior for most of gases, with only a few exceptions. ZnO-TPs show a moderate response towards NO_2_ even down to room temperature (RT). In Figure 6, the dynamic response of the sensor for 0.5 ppm of NO_2_ is reported and a *Response* = (*R_gas_* − *R_air_*)/*R_air_* ≈ 5 can be observed.

In this low temperature range MPc can also play a role. In Figure 6 the dynamic response of the ZnO-TPs+TiOPc sensor is also plotted and it is possible to see that, although response time is long, the resistivity variation is huge and a response of about 145 is obtained (29 times larger than seen in the not-functionalized sensor). On the contrary of what may be expected, TiOPc doesn't simply add an opposite p-type response to the one of ZnO-TPs, but it interacts with them to get give an enhanced n-type response.

**Figure 7.**
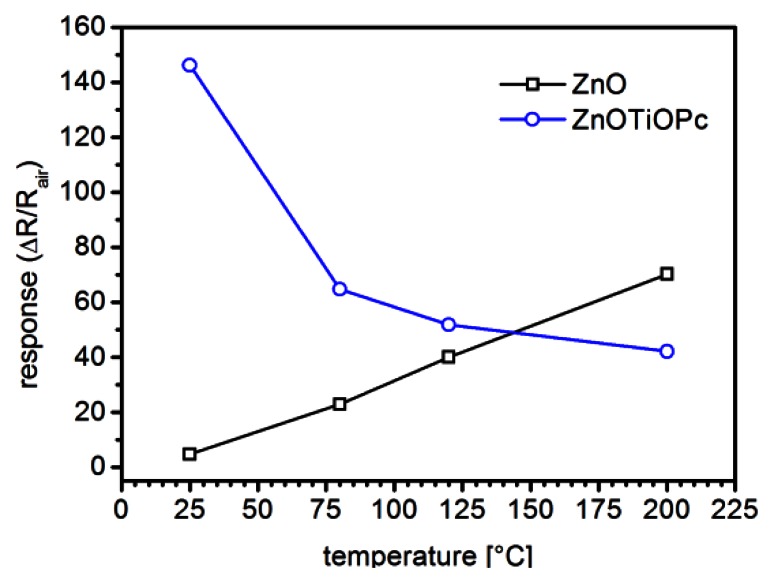
Compared responses of ZnO-TPs and ZnO-TPs+TiOPc based sensors towards 0.5 ppm of NO_2_ as a function of temperature (RH 30%).

In Figure 7 the responses of the two sensors are compared for the same NO_2_ concentration (0.5 ppm) at different temperatures ranging between RT and 200 °C. This plot clearly show that while the response of ZnO based sensors towards NO_2_ generally decreases with temperature, the hybrid ZnO-TiOPc sensor has its maximum response at the lowest tested temperature, *i.e.*, RT.

The conduction of hybrid ZnO-TPs+TiOPc still present an n-type response even at lower temperatures, showing a conduction which is mainly occurring in the ZnO nanostructures and not directly through the TiOPc film, which should show a p-type response. The TiOPc present modifies the sensitivity of the hybrid nanostructure at the lower temperatures, improving the response. The observed behavior may be ascribed to the formation of localized p-n heterojunctions where the surface of the n-type ZnO-TP is in contact with the p-type TiOPc. Indeed in the obtained hybrid ZnO-TiOPc structure, tetrapods' legs form the main percolative network through which carriers can move.

Generally surface band bending due to oxygen chemisorption induces a partial depletion of the ZnO nanostructure [18]. In the hypothesis of a p-n junction between the two materials, a further decrease in the free-carrier concentration is caused by the formation of the depleted region. This interpretation is in agreement with the observed lower conductivity of the hybrid sensor, with respect to the non-functionalized one, when exposed to air (Figure 7). At the same time the observed response enhancement can be addressed to the higher relative variation that redox reactions with the gas induce in the depleted material and also to the ability of the organic material to respond at lower temperatures.

**Figure 8.**
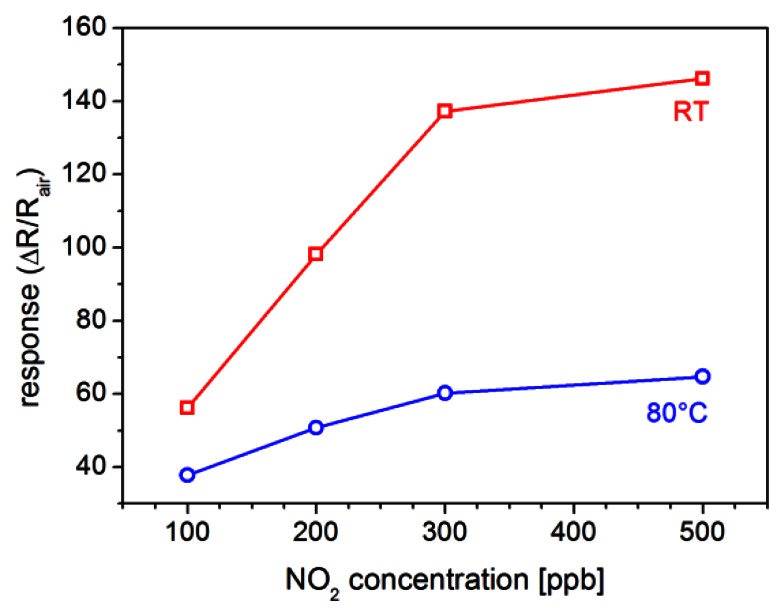
Response of a ZnO-TPs+TiOPc sensor towards different concentrations of NO_2_ at room temperature (RT) and 80 °C (RH 30%).

Sensitivity of ZnO-TPs+TiOPc sensor at low temperature can be better evaluated from the plots in Figure 8, where the response for different concentrations of NO_2_ (100, 200, 300 and 500 ppb) is reported for measurements at RT and 80 °C. This figure shows that both response and sensitivity (the first derivative of the response *vs.* concentration plot) decrease when temperature increase. The reported values are confirmed by repeating the tests several times, resulting in a good reproducibility.

**Figure 9.**
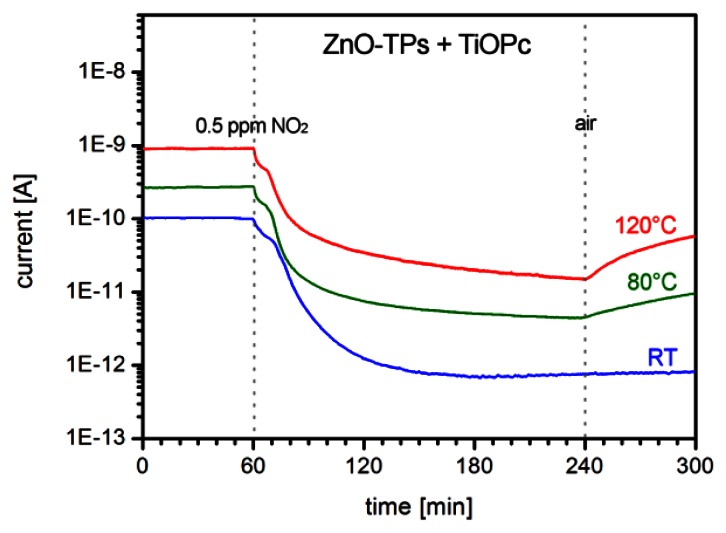
Plot of the dynamic response of a ZnO-TPs+TiOPc gas sensor towards 0.5 ppm of NO_2_ at room temperature (RT), 80 °C and 120 °C (RH 30%). Recovery time increases with temperature.

On the other hand, dynamic plots of sensor response show that the recovery time is extremely long at RT (Figure 9), even higher than the response time, and it generally takes some hours to completely recover the sensor original state once exposed to air. This is also observed in ZnO-TP only sensors, in agreement with the dominant role of ZnO-TPs in the functionalized sensor conductivity. However recovery time speed increases with temperature and the sensor can be restored in a much shorter time when heated.

Tests with other gases and vapors, like ethanol, CO, acetaldehyde and acetone did not result in visible improvements when compared with standard ZnO-TPs-based sensor in the RT-200 °C temperature range. The sensor was not tested at higher temperatures because it may affect the stability of the organic semiconductor. In conclusion, the big enhancement in the sensitivity towards NO_2_ resulted to be rather selective, as the same was not observed for other tested gases and vapors (for which the hybrid sensor showed a very low response, similar to that of non-functionalized ZnO-TPs at low temperature).

## 4. Conclusions

A nanostructured inorganic-organic hybrid based on ZnO-TPs functionalized with TiOPc has been synthesized and its sensing properties tested. Both materials are indeed used for this kind of application, but are affected by complementary issues. The behavior of the ZnO-TPs+TiOPc sensor has been studied with different gases and at different temperatures. The most evident enhancement has been observed for NO_2_: despite a slow response and recovery time, a huge response ((*R_gas_* − *R_air_*)/*R_air_* ≈ 145) has been measured for 500 ppb of NO_2_ at room temperature. At the same time the study of the response *vs.* temperature behavior revealed that sensor heating can promote a faster recovery. Higher response has been tentatively ascribed to the formation of localized p-n heterojunctions at the surface between the n-type ZnO nanostructures and the p-type TiOPc. This big enhancement in the sensitivity towards NO_2_ resulted to be rather selective, as the same was not observed for other tested gases and vapors in the studied low-temperature range (RT-200 °C).
